# Environmental Attributes Influencing the Distribution of *Burkholderia pseudomallei* in Northern Australia

**DOI:** 10.1371/journal.pone.0138953

**Published:** 2015-09-23

**Authors:** Anthony L. Baker, Jessica Ezzahir, Christopher Gardiner, Warren Shipton, Jeffrey M. Warner

**Affiliations:** 1 Environmental and Public Health Microbiology Research Group, School of Veterinary and Biomedical Sciences, James Cook University, Townsville, Australia; 2 Tasmanian Institute of Agriculture, University of Tasmania, Sandy Bay Campus, Hobart, Australia; 3 Faculty of Science, Asia-Pacific International University, MuakLek, Saraburi, Thailand; University of Toledo School of Medicine, UNITED STATES

## Abstract

Factors responsible for the spatial and temporal clustering of *Burkholderia pseudomallei* in the environment remain to be elucidated. Whilst laboratory based experiments have been performed to analyse survival of the organism in various soil types, such approaches are strongly influenced by alterations to the soil micro ecology during soil sanitisation and translocation. During the monsoonal season in Townsville, Australia, *B*. *pseudomallei* is discharged from Castle Hill (an area with a very high soil prevalence of the organism) by groundwater seeps and is washed through a nearby area where intensive sampling in the dry season has been unable to detect the organism. We undertook environmental sampling and soil and plant characterisation in both areas to ascertain physiochemical and macro-floral differences between the two sites that may affect the prevalence of *B*. *pseudomallei*. In contrast to previous studies, the presence of *B*. *pseudomallei* was correlated with a low gravimetric water content and low nutrient availability (nitrogen and sulphur) and higher exchangeable potassium in soils favouring recovery. Relatively low levels of copper, iron and zinc favoured survival. The prevalence of the organism was found to be highest under the grasses *Aristida* sp. and *Heteropogon contortus* and to a lesser extent under *Melinis repens*. The findings of this study indicate that a greater variety of factors influence the endemicity of melioidosis than has previously been reported, and suggest that biogeographical boundaries to the organisms’ distribution involve complex interactions.

## Introduction

*Burkholderia pseudomallei* is a Gram-negative saprophytic bacterium which is the causative agent of melioidosis; a clinically diverse and often fatal cause of community acquired pneumonia in tropical and subtropical regions worldwide [1]. The environmental distribution of the organism is clustered over both spatial and temporal scales and distinct biogeographical boundaries to the free dispersal of the organism have been identified [2–4]. Endemic foci of *B*. *pseudomallei* are often identified through spatial analysis of clinical cases [5, 6], however it is clear that melioidosis case clusters are prevalent throughout tropical regions of Australasia and Southeast Asia. In these regions, the highest incidence of melioidosis and the highest environmental prevalence of *B*. *pseudomallei* occurs during the monsoonal seasons when cases of melioidosis spike following high rainfall events [7]. Such events may be associated with translocation of the organism from a subterranean reservoir through mobilisation of the bacterium by groundwater [8–10].

Although much is known about melioidosis pathophysiology, a considerable gap remains regarding the natural ecology of the organism. Laboratory based comparisons of the organisms survival in different soil types are biased in that they largely ignore important considerations such as disturbance of the soil in pots and climactic effects; whilst field based seeding of *B*. *pseudomallei* is not possible due the organisms high pathogenicity. Environmental studies have indicated that there are distinct soil physiochemical attributes associated with the biogeography of the organism in the soil, including pH, iron content and water availability [11–13], however the complex nature of soil ecosystems means that only limited studies have been performed in this field. We have previously described an endemic focus of *B*. *pseudomallei* associated with Castle Hill; a granite monolith in Townsville city, northern Australia [10]. The formation represents one of three foci of clinical melioidosis in Queensland, Australia [14], and represents a unique study area in that groundwater seeps are prevalent on Castle Hill. Large quantities of viable *B*. *pseudomallei* thus are mobilised following rainfall events into a nearby area which shows a very low environmental prevalence of the organism during the dry season. This phenomenon indicates that environmental differences between the two areas likely contribute to a failure of the organism to establish in the downstream area. A detailed comparative study of the two areas therefore may hold important clues as to the small-scale environmental properties that affect the environmental establishment and persistence of *B*. *pseudomallei* in an endemic region.

This study analysed soil physiochemical properties and plant populations that may have contributed to the failure of *B*. *pseudomallei* to establish in an area regularly exposed to a drainage recharge with viable organisms. Such a study may provide valuable insight and aid in the development of an identification fingerprint for endemic regions.

## Materials and Methods

### Study Site

Consent for environmental sampling was granted by Townsville City Council. Two sites were selected based on previous sampling regimes which had identified an area of high *B*. *pseudomallei* prevalence (17.5% [95%CI 5.2–29.8]) on the piedmont slopes Castle Hill itself (19° 15.410'S; 146° 47.590'E) where groundwater seeps containing large quantities of viable organism converge into tributaries and flow into a stream [10]. For comparison, a low prevalence site on Hugh Street (19° 15.415'S; 146° 47.092'E) was identified approximately eight hundred meters downstream of the high-prevalence site where previous intensive environmental sampling was unable to detect the organism (unpublished data). Both sites are dry sclerophyll woodland bound by residential development. Wildlife activity is prevalent at the sites both of which are free from agricultural impact.

### Sample Collection

Soil samples were collected during late July and early August 2012 which is the height of the dry season in the region. Sampling was performed in the dry season to minimise any contamination of samples with *B*. *pseudomallei* from the watercourse, which flows from the high prevalence site on Castle Hill. Top-soil (n = 100) from each site was collected aseptically to 300 mm depth every meter along two parallel 50 m transects (10 m apart) into medium sterile plastic bags. In addition, soil from each bore was collected directly into pre-weighed 80 ml screw-top plastic specimen jars (Sarstedt, Germany) for gravimetric soil moisture content analysis. Transect points at which boring was not possible due to large granite outcrops or dense vegetation were relocated to the nearest sample point possible. Soil collection was performed with a hand soil auger that was washed in water then sanitised with 70% ethanol between bores. Concurrent with soil collection, plants directly above the sampling zone were identified using morphological characteristics by an experienced agronomist. Statistical analysis was performed by OpenEpi software using Chi-squared test [15].

### Soil Water Content Analysis

Gravimetric water content analysis was performed as previously described [10] using standard laboratory methods that involved weighing the soil samples prior to drying at 105°C for 24 h, cooling in a desiccator and reweighing. Calculation of gravimetric soil water content was performed as the weight difference between the original and dried samples, expressed as a percentage of the original weight of the sample. Statistical comparison of soil moisture content was performed by OpenEpi software [15] using an independent t-test.

### Cultivation and Detection of *B*. *pseudomallei* DNA

Detection of the organism utilised a real-time PCR (qPCR) assay as previously documented [2, 16]. This assay utilising a novel bacterial enrichment procedure has previously been documented to have a lower limit of detection of 5 CFU of viable *B*. *pseudomallei* per gram of soil [10]. Soil samples collected from the field were returned to the laboratory where they were stored at ambient temperature (about 25°C) overnight for immediate processing the following day. Briefly, soil samples (10 g) were transferred to 10 ml of Ashdown’s environmental isolation broth [17] containing 15 g/l tryptone (Oxoid, Australia), 5 mg/l crystal violet and 50 mg/l colistin sulphate (Sigma, Australia) in 500 ml conical Pyrex culture flasks which were sealed (the organism is facultatively anaerobic), then incubated at 37°C with agitation at 100 rpm for 24 h. Following the broth enrichment, a single use 10 μl inoculation loop (Sarstedt, Germany) was used to sub-cultivate onto Ashdown’s agar [18] supplemented with 50 mg/l colistin sulphate (Sigma, Australia). Plates were incubated aerobically at 37°C for 24 h prior to the removal of a large loop of the primary inoculum from the agar which was suspended into 50 μl of Prepman® Ultra Sample Preparation Reagent (Applied Biosystems, USA) in 1.5 ml O-ring screw-top microcentrifuge tubes (Sarstedt Germany). Samples were vigorously vortexed, then incubated in a block heater at 100°C for 10 min. Samples were centrifuged at 16,000 × g for two min and the supernatant removed to new 1.5 ml O-ring screw-top microcentrifuge tubes to be used as template for qPCR. No template controls were performed in triplicate utilising Prepman® Ultra Sample Preparation Reagent (Applied Biosystems, USA) without the addition of bacterial inoculum and were processed as for the other samples.

Real time PCR targeted a 115-base-pair region within *orf2* of the type III secretion system of *B*. *pseudomallei* [16] on a Rotor-Gene 6000 series thermocycler (Corbett Life Science, Australia) as previously described [2]. The assay was previously determined to be significantly more sensitive than cultivation-based techniques, with no evidence of false-positive results [12]. Reactions were formulated to 20 μl and consisted of 1 × GoTaq Colourless Master Mix (Promega, Australia), 256 nM of FAM-BHQ labelled probe (BpTT4208P: 5’-FAM-CCGGAATCTGGATCACCACCACTTTCC-BHQ-3‘), 400 nM of each primer (BpTT4176F: 5’-CGTCTCTATACTGTCGAGCAATCG-3’ and BpTT4290R: 5’-CGTGCACACCGGTCAGTATC-3’) and molecular biology grade H_2_O (Sigma, Australia) to 20 μl. Template was 1 μl of Prepman® Ultra Sample Preparation Reagent as previously prepared (including DNA extraction controls) or molecular biology grade H_2_O (Sigma, Australia) for qPCR no template controls. Cycling comprised an initial denaturation period of 3 min at 95°C, followed by 45 cycles of 95°C for 15 s and 59°C for 15 s. Cultivation, DNA preparation and qPCRs were performed in duplicate on all samples. A *B*. *pseudomallei* clinical isolate, C3 was used as positive control.

Samples reactive by qPCR were sub-cultivated in triplicate onto Ashdown’s agar [18] supplemented with 50 mg/l colistin sulphate (Sigma, Australia) for five days at 37°C and examined daily for the presence of typical *B*. *pseudomallei* colonies. Presumptive *B*. *pseudomallei* colonies were subcultured onto Ashdown’s agar and identified by qPCR using the assay as described.

### Soil Physiochemical Analysis

Soil samples were grouped for physiochemical analysis into three treatment groups; low-prevalence site (none of which tested qPCR positive for *B*. *pseudomallei*), high prevalence site testing qPCR positive for *B*. *pseudomallei* and high-prevalence site testing qPCR negative for *B*. *pseudomallei*. Each treatment group (n = 3) consisted of two 500 g soil samples pooled from five different but individual soil samples of 100 g selected at random. Pooled soil samples (n = 6) were thoroughly mixed by shaking prior to physiochemical analysis which was performed by a NATA-registered (National Association of Testing Authorities) and ASPAC-affiliated (Australasian Soil and Plant Analysis Council) laboratory run by Incitec Pivot Limited. Statistical analysis of the raw data was performed using the two-tail t-test function in Microsoft Excel version 14.4.3 (Microsoft, USA).

## Results

*Burkholderia pseudomallei* DNA was detected by qPCR in 14 of 200 (7% [95%CI 3.88–11.47]) of all the pre-enriched soil samples tested. No *B*. *pseudomallei* was detected in the low-prevalence site. All qPCR positive samples originated from the high-prevalence site at Castle Hill; a prevalence of 14% (95%CI 7.87–22.27) indicating a significant difference between sites (p = 0.000). Cultivation of the qPCR positive enriched broth samples on triplicate agar plates yielded five culture positive samples, a lower sensitivity, yet not of significant difference to those obtained using qPCR.

In general, soils from both sites were nutritionally deficient (Table 1) with very low nitrogen, organic carbon and cation exchange capacity (CEC). Additionally, soil from both sites had a high sand content (low-prevalence 81%; high-prevalence 74.25%; [2–0.02mm particle size]), with insignificant differences between sites. Independent t-test determined that mean soil water content between the sites was significantly different (p < 0.001), with 15.7% (SD 11.05) gravimetric water content at the low-prevalence site and 7.4% (SD 1.95) gravimetric water content at the high-prevalence site. Soil pH from both sites was mildly acidic, an independent t-test identified statistically significant variances (p < 0.005) between the low-prevalence site with a mean pH of 5.65 (SD 0.071) and the high-prevalence site with a mean pH of 6.08 (SD 0.084).

**Table 1:** Duplicate dry season soil physiochemical attributes from a site with a high-prevalence of *Burkholderia pseudomallei* (qPCR +ve soil samples vs. qPCR-ve soil samples) and a site with a low-prevalence of the organism (all qPCR-ve soil samples), which is inundated regularly with groundwater containing a large quantity of viable *Burkholderia pseudomallei*. Statistically significant p-values are identified in bold text.

**Site**	**Acidity, Alkalinity, Salinity**			**Micronutrients (DTPA)**			
	pH	Electrical conductivity (dS/m)	Chloride (mg/kg)	Copper (mg/kg)	Iron (mg/kg)	Manganese (mg/kg)	Zinc (mg/kg)
**Low-prevalence 1**	5.6	0.06	17	5	180	10	43
**Low-prevalence 2**	5.7	0.06	21	3.8	140	7	36
**Low-prevalence mean**	5.65	0.06	19	4.4	160	8.5	39.5
**High-prevalence +ve 1**	6.1	0.01	18	0.1	10	15	1.3
**High-prevalence +ve 2**	6	0.01	16	0.1	8	12	1.1
**High-prevalence-ve 1**	6.1	0.02	16	0.3	17	20	12
**High-prevalence-ve 2**	6	0.01	11	0.4	16	19	6.1
**High-prevalence mean**	6.05	0.0125	15.25	0.225	12.75	16.5	5.125
**t-test (High-prevalence vs Low-prevalence)**	**0.0003**	**0.0000**	0.1498	**0.0001**	**0.0000**	0.0248	**0.0003**
	**Macronutrients**						
	Organic Carbon (%)	Nitrate Nitrogen (mg/kg)	Available Phosphorous (BSES) (mg/kg)	Available Phosphorous (Colwell) (mg/kg)	Phosphorous Buffer Index	Available Potassium (Colwell) (mg/kg)	Sulphate Sulphur (mg/kg)
**Low-prevalence 1**	1.4	7	28	8	58	44	21
**Low-prevalence 2**	1.4	7	25	6	47	44	20
**Low-prevalence mean**	1.4	7	26.5	7	52.5	44	20.5
**High-prevalence +ve 1**	0.5	1	7	<5	41	93	2
**High-prevalence +ve 2**	0.7	1	6	<5	50	89	3
**High-prevalence-ve 1**	1	3	18	8	50	150	2
**High-prevalence-ve 2**	0.8	2	8	11	48	110	3
**High-prevalence mean**	0.75	1.75	9.75	9.5	47.25	110.5	2.5
**t-test (High-prevalence vs Low-prevalence)**	**0.0048**	**0.0004**	0.0062	0.1597	0.2481	0.0143	**0.0000**
	**Exchangeable Cations (Proportions)**						
	Cation Exchange Capacity (meq/100g)	Exchangeable Calcium (%)	Exchangeable Magnesium (%)	Exchangeable Potassium (%)	Exchangeable Sodium (%)	Exchangeable Aluminium (%)	Ca:Mg ratio (%)
**Low-prevalence 1**	4.1	71	22	2	3	2	3.2
**Low-prevalence 2**	3.9	71	21	3	3	3	3.4
**Low-prevalence mean**	4	71	21.5	2.5	3	2.5	3.3
**High-prevalence +ve 1**	2.2	30	45	8	2	15	0.7
**High-prevalence +ve 2**	2.6	25	47	6	2	19	0.5
**High-prevalence-ve 1**	3.4	58	29	10	1	3	2
**High-prevalence-ve 2**	2.7	44	37	8	1	10	1.2
**High-prevalence mean**	2.725	39.25	39.5	8	1.5	11.75	1.1
**t-test (High-prevalence vs Low-prevalence)**	0.0115	0.0216	0.0198	**0.0040**	0.0102	0.0938	**0.0040**
	**Particle Size (%)**						
	Course sand (2–0.2mm)	Fine sand (0.2–0.02mm)	Silt (0.02–0.002mm)	Clay (<0.002mm)			
**Low-prevalence 1**	41	39	16	4			
**Low-prevalence 2**	49	33	15	3			
**Low-prevalence mean**	45	36	15.5	3.5			
**High-prevalence +ve 1**	38	36	19	8			
**High-prevalence +ve 2**	39	34	20	8			
**High-prevalence-ve 1**	42	33	18	8			
**High-prevalence-ve 2**	38	37	18	8			
**High-prevalence mean**	39.25	35	18.75	8			
**t-test (High-prevalence vs Low-prevalence)**	0.0655	0.6350	**0.0048**	**0.0000**			

No significant physiochemical differences were identified between soil samples from the high-prevalence site that tested qPCR positive for the organism against those that did not (p = 0.01), however several significant differences emerged between the high-prevalence site and the low-prevalence site downstream (Table 1). Significant differences (p < 0.001) were identified in electrical conductivity (low-prevalence 0.06; high-prevalence 0.014; [ds/m]), nitrate/nitrogen (low-prevalence 7; high-prevalence 1.8; [mg/kg]), sulphate/sulphur (low-prevalence 20.5; high-prevalence 2.4 [mg/kg]), copper (low-prevalence 4.4; high-prevalence 0.24 [mg/kg]), iron (low-prevalence 160; high-prevalence 13 [mg/kg]), zinc (low-prevalence 40; high-prevalence 4.8 [mg/kg]) and exchangeable potassium (low-prevalence 2.5%; high-prevalence 8.2%). No significant differences were identified for chloride, organic carbon, phosphorus, potassium, manganese or exchangeable cations.

Chi-squared test indicated a distinct trend between the presence of the organism in soil directly beneath grass species of the family Poaceae (Table 2) and its absence under broadleaf plants (Laminaceae and Fabaceae) and sedge (Cyperaceae). An Aristida species and Black Spear Grass, *Heteropogon contortus*, were the predominant species associated with qPCR positive soil samples (57% [95%CI 28.86–82.34]).

**Table 2:** Prevalence of *Burkholderia pseudomallei* recovered from directly beneath various plant species from the high prevalence site only.

Species	Family	Samples (*n*)	*Burkholderia pseudomallei* +ve	Percentage +ve	95% CI
*Aristida* sp.	Poaceae	9	2	22	2.8–60
*Heteropogon contortus*	Poaceae	46	8	17	7–31
Melinis repens*	Poaceae	30	3	10	2.1–26
*Cymbopogon* sp.	Poaceae	1	0	0	0–97
*Hyptis* sp.*	Lamiaceae	3	0	0	0–70
*Stylosanthes* sp.*	Fabaceae	4	0	0	0–60
*Cyperus* sp.	Cyperaceae	5	1	20	0.5–71

Exotic species are marked with an asterisk.

## Discussion

This study compared the physiochemical properties of an environment exposed to a high quantity of viable *B*. *pseudomallei* during the wet season in which the organism has failed to establish a population. The prevalence of *B*. *pseudomallei* DNA in broth-enriched soil from Castle Hill (high prevalence site) was in the range of our previous work at the same site, with a prevalence of 14% recorded during this study and 17.5% from our previous work during the 2010 dry season [10]. Whilst it is possible that assay bias has occurred due to differences in soil biophysiochemical properties between the high and low prevalence site, previous seeding experiments into soil from the low-prevalence site have not provided evidence of assay inhibition. Despite large quantities of viable *B*. *pseudomallei* flowing into the low-prevalence site during the wet season [10], the organism does not appear to have established at the site. Given similarities in climatic parameters between the sites it is highly probable that soil from the low-prevalence site is not conducive to one of establishment, proliferation or persistence of the organism in a cultural state. The soils may contain a factor(s) that represents an as yet undefined biogeographical boundary.

Significant differences were not identified between qPCR reactive soil samples and qPCR non-reactive soil samples from Castle Hill suggesting that micro-spatial distribution of the organism in the soil may be dependent on physical or biological factors other than those examined over the course of this study. Soils from all treatments were nutritionally deficient with very low nitrogen, organic carbon and CEC. The CEC is generally considered an indicator of soil fertility as it relates to the soil’s ability to hold cations, typically held by colloids such as humus (organic carbon) and clay. The very low CEC of soils on Castle Hill (~2.7) and high percentage sand indicate a very nutritionally poor soil. The high prevalence of the organism in these soils correlates with the extreme hardiness of *B*. *pseudomallei* as identified by its ability to survive in nutritionally poor environments for decades [19]. This may indicate an adaptation to nutritionally poor environmental niches.

Previously, the presence of *B*. *pseudomallei* in soil samples has been associated predominantly with clay loam soil, a combination of roughly equal quantities of clay, silt and sand [8, 12]. The results of this study however, indicate that soil types predominantly found on and around Castle Hill are around 75% sand with less than 10% clay, and containing the clay fractions kaolinite with traces of illite.

A high moisture content (greater than 10–15–40%) has been implicated as important in enhancing the presence or survival of *B*. *pseudomallei* in soil [11, 20–22]. Larsen and colleagues (2013) showed that prolonged survival was possible in desiccated (91 days) and intermittently irrigated soils (113 days) where the moisture content varied from close to 0% (after 14 days starting at ~8%) for the desiccated soil to approximately 5–13% in the intermittently irrigated soil. Whereas survival was significantly (p < 0.05) higher in the intermittently irrigated soil for the greater part of the study, recovery at 70 days was identical in both soils. Larsen *et al*. explained the apparent conflict with previous findings on the basis of variability in environmental isolate behaviour. However, their findings do not relate well to field situations in that sterilised soil was used in their trials. The findings of our study suggest that soil moisture content may play a role, yet indicate that higher soil moisture content may not necessarily make an outstanding contribution to survival of the organism during the dry season. Further investigation of this aspect would need to incorporate measurements of water availability (potential) and include the possible protective effects of the clay fraction on survival especially in situations experiencing relatively rapid drying episodes [23]. It has been postulated that well drained soils are not conducive to survival of the organism given patterns in the incidence of melioidosis in northern Australia [6]. However, the results of the present study indicate that perhaps fluctuations in soil water content and/or the seasonal rise and fall of the water table are more important determinants of the organism’s presence. Such a hypothesis may help to explain the distribution of clinical melioidosis, which in Queensland is more often associated with tropical regions that exhibit a well defined wet and dry season [14]. It appears to be supported by a recent study indicating that low water holding capacity soil may be better adapted to support *B*. *pseudomallei* than higher water holding capacity sites [13]. This suggests that other factors alter the microenvironment to enhance survival under desiccating conditions. Two such factors have been suggested with other bacteria, namely extracellular polysaccharide production and association of bacterial cells with clay minerals [24, 25]. Both the formation of an extracellular matrix and clay envelopes slow the rate of drying and a polysaccharide matrix functions to retain higher water content and perhaps facilitate increased nutrient availability in bacterial colonies.

The formation of clay protective associations may have been a factor in our studies. Montmorillonite appears to be effective in enhancing survival of a number of soil bacteria under desiccating conditions but other clay minerals such as illite and kaolinite can exert a beneficial effect, which is bacterium-specific [26, 27]. Detailed studies involving *B*. *pseudomallei* survival in relation to clay mineral soil content over multiple sites may be merited. Such studies would be particularly valuable if complemented by laboratory studies using a number of clay minerals in various environmental configurations. The microhabitat generated by clay minerals is significant to microbial behaviour [24].

*Burkholderia pseudomallei* is a well-known capsular polysaccharide producer, which classically has been associated with virulence [28]. The general opinion is that capsular material gives a survival advantage under desiccating conditions [29, 30]. Pseudomonas colonies subjected to desiccation in a nutrient poor soil while growing showed a heightened ability to produce extrapolysaccaride compared to those functioning at a high water potential [25]. It may be rewarding to investigate interactions among factors in the environment which stimulate capsule formation in *B*. *pseudomallei* and establish the survival advantages conferred.

It has also been suggested that organic carbon in soil may support the survival of the organism during periods of low water availability [11]. However, the low organic carbon levels from our high-prevalence and low water content site does not lend support to this idea. Clearly, additional work utilising water potential parameters, giving attention to soil nutritional status and considering the dynamics of other microbiota members needs to be investigated in order to gain a better understanding of the complex interactions occurring.

Some previous studies have indicated that a high availability of iron (Fe) may be an important determinant of *B*. *pseudomallei* endemicity [9, 12, 31]. The later study used autoclaved soil and did not record the contaminant level of iron in the experimental soil sample to which high levels of iron were added in the experimental set-up. This means that little useful information for field application can be gained. The Draper et al. study investigated bore water sites and correlated recovery of *B*. *pseudomallei* with levels of iron of 2 and 4 mg/l in the dry and wet season respectively. Indeed, all clinical strains (84) collected in one study showed siderophore production and it was not surprising the organism showed a markedly higher growth rate on growth media supplemented with iron compared to growth on unaltered media [32]. In the present study a significant difference existed between the iron content in the high and low-prevalence areas. However, iron levels on the positive recovery Castle Hill site were significantly lower than those in the low prevalence site, with a mean of 12.75 mg/kg. Even this level is six fold higher than the levels recorded by Draper *et al*. as supporting survival of the bacterium.

*Burkholderia pseudomallei* was associated most frequently with *Aristida* sp. and *Heteropogon contortus* (Black Spear Grass) and to a lesser extent with *Melinis repens* (Natal Grass). Differences in the macrofloral composition between the two sites is evident. Whilst these differences may impact *B*. *pseudomallei* persistence, a previous study of 50 *H*. *contortus* samples from the low-prevalence site failed to detect *B*. *pseudomallei* (unpublished data). As this species was significantly correlated with the presence of *B*. *pseudomallei* on Castle Hill, it indicates that additional environmental factors are at likely at work. Previous studies from the Northern Territory of Australia have found that the organism was significantly associated with annual *Sorghum* sp. [12]. More recently, the organism has demonstrated a capability to associate with and even infect scrubs and grasses [33–35]. Native and introduced grass species in northern Australia were found by Kaestli *et al*. to support the presence of *B*. *pseudomallei* in the rhizosphere. These included *Brachiaria humidicola* (Tully Grass), *Pennisetum pedicellatum* (Desho Grass) and *Pennisetum* polystachion (Mission grass), *Paspalum plicatulum* (Brownseed Paspalum), *Sorghum intrans* and *Oryza rufipogon* (Wild Rice). Association was not restricted to the rhizosphere but extended to the leaves of some plants and even internal colonisation was detected in Mission and Tully grasses. Although *B*. *pseudomallei* has been associated with a number of plant species, the extent of these relationships and diversity of plant associations remain to be fully investigated, yet indicate a significant survival strategy. The rhizosphere is a zone of intense microbial activity and location in this zone is significant to the survival and life cycle events of a number of microbes. Both cooperative and antagonistic influences may be experienced. However, very few details are known about the activities of *B*. *pseudomallei* in this region. Indicative antagonistic effects on the bacterium have been noted from a number of related Burkholderia species isolated from agricultural soils [36, 37].

Although relatively few studies have examined the physiochemical attributes of *B*. *pseudomallei* endemicity, conflicting reports from multiple regions suggest that complex interactions among climate [7], soil physiochemistry [13, 38], topography [10] and macro-flora [12] are important determinants of melioidosis endemicity. The scene is complicated even further by bacterium strain differences.
